# Should developmental coordination disorder be diagnosed before the age of 5 years?

**DOI:** 10.1111/dmcn.16389

**Published:** 2025-06-20

**Authors:** Marina M. Schoemaker

**Affiliations:** ^1^ University of Groningen, University Medical Center Groningen, Department of Human Movement Sciences Groningen the Netherlands

## Abstract

**This commentary is on the original article by De Roubaix et al. on pages** 1601–1615 **of this issue.**

One of the clinical practice recommendations for children with developmental coordination disorder (DCD) of the European Academy of Childhood‐onset Disability (EACD) is not to make a formal diagnosis of DCD under the age of 5 years, unless there are severe motor problems.^1^ The rationale behind this recommendation is the intra‐individual variability of motor performance before the age of 5 years. Two follow‐up studies support this variability.^2^, ^3^ In both studies, preschool children at risk for DCD were included, defined by scores below respectively the 16th and 10th percentile on the Movement Assessment Battery for Children, Second Edition (MABC‐2),^4^ and followed for 2 years. After 2 years, 50% of the children still performed in the clinical range, but 50% improved their motor performance to typical standards. Several factors may have contributed to this improvement.

First, assessing preschool children is challenging, as children may lack the attention and motivation to perform optimally. Their performance does not necessarily reflect what they can do. Second, their level of performance is driven by the opportunities for motor learning the children had. Differences in child‐rearing practices may lead to differences in exposure to motor learning opportunities, leading to different levels of motor task performance. After the age of 3 years, formal schooling starts, often offering more opportunities for motor learning, and enabling children to catch up who lacked behind in motor skills due to insufficient practice.

However, both follow‐up studies included community samples, only categorizing children as at risk for DCD by low scores on the MABC‐2.^2^, ^3^ Longitudinal follow‐up studies including children at risk for DCD were lacking. The study by De Roubaix et al. fills this gap.^5^ In this study, children with developmental concerns, children born preterm or with low birthweight, and children with severe neonatal complications were assessed by a multidisciplinary team to determine whether they were at risk for DCD, by combining findings from both standardized motor assessments, anamnesis, and observation of movement quality. After 2 years, 85% of the children who were classified as at risk for DCD at 3 years were still diagnosed as DCD or at risk for DCD. This implies that intra‐individual stability of motor performance is much higher in high‐risk young children. This is an important result, as it highlights that children with motor challenges in high‐risk populations can already be identified at 3 years of age. The results also stress the importance of multiple assessments for identification of DCD at preschool age, as the outcome at follow‐up was more stable compared to the follow‐up studies in which only one motor test was used.^2^, ^3^

However, the classification at risk for DCD was based upon clinical judgement and the assessment involved a non‐standardized assessment of quality of movement, impacting the generalizability of the results to other clinical practices. This calls for the need to develop standardized assessment instruments for quality of movement. In addition, to advance the early identification of children at risk for DCD it would be helpful to objectify clinical decision making by creating clinical algorithms, which consist of stepwise procedures that can support clinicians in making decisions about a diagnosis. To enable this, more follow‐up studies such as the De Roubaix et al. study are needed to develop such an algorithm.^5^

Should DCD be diagnosed under the age of 5 years? The De Roubaix et al. study teaches us that in high‐risk pre‐school children in particular, clinicians should be aware of the risk of DCD, and in case of concerns use multiple assessments to assess whether these children meet the diagnostic criteria for DCD. Early identification may prevent secondary consequences by offering early intervention.

## Data Availability

Not required.
